# Analysis of the Spatial Association Network of PM_2.5_ and Its Influencing Factors in China

**DOI:** 10.3390/ijerph191912753

**Published:** 2022-10-05

**Authors:** Huiping Wang, Qi Ge

**Affiliations:** Western Collaborative Innovation Research Center for Energy Economy and Regional Development, Xi’an University of Finance and Economics, Xi’an 710100, China

**Keywords:** PM_2.5_, spatial association network, social network analysis, block model

## Abstract

The spatial association network of PM_2.5_ is constructed using a modified gravity model, with the data of 31 provinces in China from 2009–2020. On this basis, the spatial correlation structure of PM_2.5_ and its influencing factors were investigated through social network analysis (SNA). The results showed that, first, the PM_2.5_ has a typical and complex spatial correlation, and the correlation degree tends to decrease with the implementation of collaborative management. Second, they show that there is a clear “core-edge” distribution pattern in the network. Some areas with serious PM_2.5_ pollution have experienced different degrees of decline in centrality due to policy pressure. Third, the network is divided into “net benefits”, “net spillovers”, “two-way spillovers” and “brokers”. The linkage effect among the four blocks is obvious. Fourth, the government intervention and the industrial structure differentiation promote the formation of the network, but environmental regulation and car ownership differentiation have the opposite effect on the network.

## 1. Introduction

In recent decades, China has made remarkable achievements in economic development. However, due to the rapid economic development and urbanization, as well as the lack of atmospheric environmental protection, China is facing serious air pollution problems [1,2,3,4]. According to the China Environment Bulletin, among all the polluted days, PM_2.5_ constitutes 60% of them, and China is one of the countries with high and rapidly growing PM_2.5_ concentrations, worldwide [5]. In China, PM_2.5_ has already had serious negative impacts on residents’ health, economic development, transportation and travel, agricultural production, and on the country’s international image [6,7,8,9,10]. Therefore, controlling PM_2.5_ concentrations is important for China in order to reconcile the contradiction between economic development and environmental pollution [11]. To improve air quality and mitigate the PM_2.5_ pollution problem, several measures have been adopted by the Chinese government to combat air pollution, and several research projects have been initiated to investigate the causes of air pollution [12]. However, despite the significant reduction in PM_2.5_ concentrations in recent years, there is still a large gap between the overall level of air quality in China and that of other developed countries. Additionally, the situation is still severe, given the frequent occurrence of heavy pollution events and the co-pollution of PM_2.5_ and the ozone. PM_2.5_ management remains a common concern for governments at all levels and for academia [13,14].

Regarding PM_2.5_ management, early studies have focused on source analysis—mostly analyzing the generation process and the chemical composition of PM_2.5_ from physical and chemical perspectives, considering industrial emissions, coal combustion, dust and motor vehicle exhaust as important sources of PM_2.5_. Thus, these studies have generally proposed solution ideas from a natural science perspective [15,16,17]. Subsequently, some scholars have explored the socioeconomic drivers of PM_2.5_, such as the economic development, urbanization, energy consumption, industrial structure, transportation structure, and vegetation cover, thus, arguing for the development of relevant pollution management policies, starting from an economic development approach [18,19,20,21,22,23,24]. Due to the accelerated regional integration process and increasingly frequent economic activities, the PM_2.5_ in one region affects the PM_2.5_ in other regions, exhibiting certain spatial correlations and clustering characteristics. That is, PM_2.5_ pollution in one region will aggravate PM_2.5_ pollution in the surrounding areas. Therefore, it is more important for pollution management to consider the influence of spatial factors and strengthen the synergistic cooperation between regions [25,26,27,28,29].

Although existing studies have been conducted in terms of PM_2.5_ source analysis, natural and social influences, and the characteristics of the spatial and temporal distribution of PM_2.5_—such as using spatial statistical methods and spatial econometric models to examine the spatial clustering characteristics and spillover effects of PM_2.5_ pollution—there are still some limitations in research on the spatial association relationships of PM_2.5_.

Although existing studies have analyzed the sources of PM_2.5_ using ordinary least squares; generalized moment estimation; geographically weighted regression model, and have used location entropy; gravity-center models; standard deviational ellipse models; spatial statistical methods; and spatial econometric models to test the spatial aggregation characteristics and spillover effects of PM_2.5_ pollution, there are still some limitations in the research on the spatial correlation of PM_2.5_. First, most existing studies have been based on attribute data rather than relational data. Attribute data make it difficult to portray the overall spatial association characteristics of PM_2.5_. In contrast, relational data often determine the performance of attribute data and are more valuable for analysis [30]. Second, existing studies do not further reveal the spatial structural pattern and spatial clustering of PM_2.5_, and the traditional measurement methods can only examine quantitative effects based on the consideration of spatial factors, they cannot reveal the effects of spatial association. In recent years, as a new method of analyzing the spatial association of variables, social network analysis (SNA) has been applied in a variety of fields, including economics, management, and computer science, obtaining rich results [31,32,33,34]. SNA is an interdisciplinary analysis method for exploring the overall characteristics, individual characteristics, and the structural characteristics of spatial relational networks [35,36]. SNA does not reveal network association characteristics solely based on attribute data, but also reveals connections between network nodes via relational data. Meanwhile, with the help of the QAP in SNA, the influence of other correlations and regional differences on air pollution can be studied. Recently, scholars have increasingly used SNA to examine the complex network relationships of regional environmental pollution from the perspective of relational data, such as the correlations between environmental pollution investment in China [37] and atmospheric pollution in the Yangtze River Delta region [38].

To achieve the quantitative targets of air pollution reduction in each region of China during the 14th five-year plan period, it is necessary to consider the PM_2.5_ situation in each region and the causal relationship and synergistic control of air pollution between the regions. Therefore, investigating the characteristics of the spatial association network of PM_2.5_ and the influencing factors of the network holds great practical significance for improving the cross-regional collaborative PM_2.5_ control mechanism and in helping decision makers allocate emission responsibilities. This paper made the following three contributions:

(1) This paper took 31 Chinese provinces as its research objects and explored the association network structure of PM_2.5_ among the provinces from a more macroscopic perspective, providing a new idea for research on collaborative PM_2.5_ governance.
(2) An improved gravity model was applied to identify the spatial association network of PM_2.5_ among the 31 provinces, and the structural patterns of the network were identified. The characteristics of the network were analyzed in detail at three levels, i.e., the overall, individual, and subgroup levels, which revealed the role played by each region in the network.
(3) The block model was used to reveal the spatial clustering of PM_2.5_ and the influencing factors of the network were empirically examined.

The rest of this paper is organized as follows: Section 2 presents the materials and methods, Section 3 presents the results and discussion, and the conclusions are presented in Section 4.

## 2. Materials and Methods

### 2.1. Improved Gravitational Model

In SNA, the relationships between social actors serve as the basic quantitative unit of analysis. Furthermore, by using algebraic methods and graph theory tools, the method describes a network’s relationship patterns as well as its overall characteristics, individual characteristics, and structural characteristics. Therefore, determining the relationships is the key to SNA. Based on the literature, the current methods for describing spatially related relationships are mainly based on the gravity models. This paper adopts an improved gravity model, with the IPAT model, to construct the spatial association network of PM_2.5_ in China. In the IPAT model [39], I = P × A × T. Where I is the environmental load, P is the population, A is the economic development, and T is the technological level. The IPAT model suggests that environmental pollution is mainly influenced by the following three factors: population, economy, and technology. On this basis, the improved gravitational model used in this study was as follows:

(1) $$y_{ij}=k_{ij}\frac{\sqrt[{4}]{G_{i}P_{i}T_{i}C_{i}}\sqrt[{4}]{G_{j}P_{j}T_{j}C_{j}}}{D_{ij}^{2}},k_{ij}=\frac{C_{i}}{C_{i}+C_{j}}$$

where i and j represent different provinces; $y_{ij}$ characterizes the gravitational force between province i and province j in regard to PM_2.5_ pollution; $k_{ij}$ characterizes the contribution of province i to the PM_2.5_ pollution between provinces i and j; $D_{ij}$ denotes the geographical distance (GD) between province i and province j, and is replaced by the distance between provincial capitals. $P_{i}$ and $P_{j}$ denote the total population; $T_{i}$ and $T_{j}$ denote the technological level; $C_{i}$ and $C_{j}$ denote the PM_2.5_ pollution; $G_{i}$ and $G_{j}$ denote the gross regional product. Based on Equation (1), the gravitational matrix was derived, and the average of each row of the gravitational matrix was taken as the critical value. Gravitational force above the critical value was recorded as 1, which meant that the province in the row had a PM_2.5_ association with the province in the column. Conversely, if the gravitational force was lower than the critical value in the row, it was recorded as 0, which meant that the province in the row did not have a PM_2.5_ association with the province in the column. Thus, the binary matrix was constructed.

### 2.2. Indicators of the Spatial Association Network

#### 2.2.1. Overall Network Characteristics

The overall network characteristics were described by the following four dimensions: the network association degree, network density, network rank degree, and network efficiency. The network association degree reflected the accessibility of the spatial association network, i.e., whether any two nodes in it could be connected to each other. The fewer provinces that were reachable from the network, the smaller the degree of network association. Based on the ratio of the number of actual correlations to the maximum number of relationships, the network density was calculated, which was an indicator of the closeness of the connection between nodes. The higher the network density was, the stronger the pollution links among provinces in the network. The network rank degree refers to how asymmetrically accessible network nodes were to each other. The larger the rank degree was, the higher the number of unidirectional relationships and the greater the extent to which the hierarchical structure of PM_2.5_ emissions among provinces was distinct. The network efficiency was determined by how many redundant connections it had, given the number of constituents it contained. As the number of connections increases in a network, so does its stability and efficiency.

#### 2.2.2. Individual Network Characteristics

In SNA, degree centrality (DC), betweenness centrality (BC), and closeness centrality (CC) are generally used to portray individual network characteristics.

DC can be used to measure the extent to which each province is at the center of the network, based on the number of connections. In the directed network, DC is also divided into out-degree (Out) and in-degree (Ind) centrality. The former refers to the number of associations actively sent by a province to other provinces, and the latter refers to the number of associations passively received by a province to other provinces. DC can be calculated using Equation (2), where n is the number of nodes, the same applies below.

(2) $$DC=\frac{\left(Ind_{i}+Out_{i}\right)}{2(n-1)}$$

BC is a measure of the degree to which a province is able to control the interrelationships among other provinces, or how “in the middle” of other provinces it is. A higher BC means that the more a province controls the interactions between other provinces, the more the province is at the center of the network. In Equation (3), BC was calculated, where $g_{jk}(i)$ denoted the number of shortest association paths between nodes j and k through node i; $g_{jk}$ denotes the number of all shortest association paths between nodes j and k, where k≠i≠j and j<k.

(3) $$BC=\frac{2\sum_{j}^{n}\sum_{k}^{n}g_{jk}(i)/g_{jk}}{n^{2}-3n+2}$$

CC measures how much a province in a network is independent of other provinces in the pollution linkage process. The higher a province’s closeness to the center, the more direct connections exist between the province and other provinces, with the province being the central actor in the network. CC can be calculated using Equation (4), where $d_{ij}$ denotes the shortcut distance between two nodes, as follows:

(4) $$CC=\frac{\sum_{j=1}^{n}d_{ij}}{n-1}$$

#### 2.2.3. Spatial Clustering Analysis

In cluster analysis, the block model is one of the most commonly used methods. In the model, the role of each location (block) in a network can be analyzed. The roles of blocks in spatially connected networks are usually classified as four types. The first is the net benefit role, where this type of block receives both ties from other types of blocks and ties from this type of block. This type of block receives significantly more ties from other types of blocks, than it spills over to other types of blocks. The second is the net spillover role. In contrast to receiving ties from other blocks, this type of block sends out very few. The third is the two-way spillover role, where this type of block both sends ties and receives ties from other types of blocks. It receives more ties from within blocks belonging to this type of block. The fourth is the broker role, where this type of block both sends ties and receives ties from other blocks. There are more ties between this type of block and blocks belonging to other types of blocks.

#### 2.2.4. QAP Analysis

From a quantitative perspective, since the relational data used in this paper were linkage data, they directly contradicted the principle of covariance. The QAP method compares the similarity of two matrices, gives the correlation coefficients between the two matrices, and performs a nonparametric test on the coefficients. The basic regression model in QAP regression analysis is shown in Equation (5), where Y is the explained variable and X is the explanatory variable. In this paper, Y denoted the spatial correlation matrix of PM_2.5_, and $X_{i}$ denoted the matrix of influencing factors of the correlations, as follows:

(5) $$Y=f(X_{1},X_{2},\cdots \cdots X_{n})$$

### 2.3. Data Sources

The sample period of this study that was selected was from 2009 to 2020, and 31 provinces (excluding Hong Kong, Macao, and Taiwan) were used as network nodes. The data sources and the data processing were as follows: the GDP of each province was deflated, based on the base period of 2009, to eliminate the effect of price factors. The government intervention was measured by the share of fiscal expenditure in GDP. By measuring the value added of the secondary industry to GDP, the industrial structure was determined. The environmental regulation can be measured by the amount invested in pollution control. The China Statistical Yearbook provided the data. The number of patents granted was from the Patent Cloud database.

## 3. Results and Discussion

### 3.1. Characteristics of the Overall Network Structure

**Figure 1:**
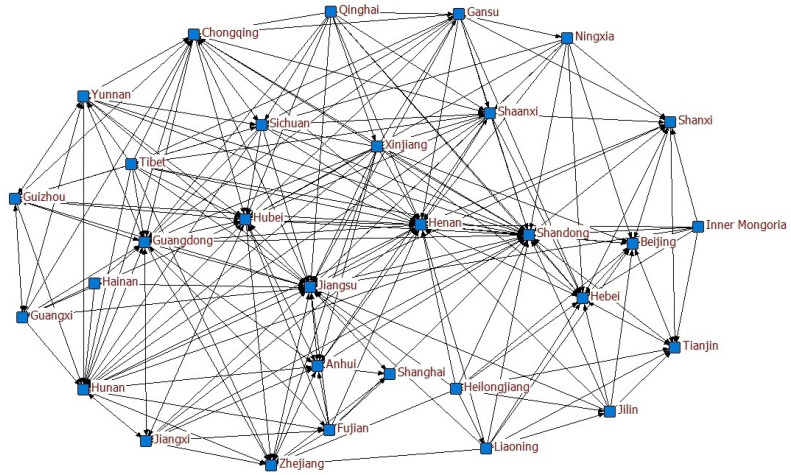
Spatial association network of PM_2.5_ in China in 2020.

We determined the spatial association network of PM_2.5_ in China from 2009 to 2020, using an improved gravity model, and established the relationship matrix. The network map, based on the relationship data, not only showed the spatial correlation of PM_2.5_ among the provinces but was also used to examine the degree of spatial correlation at the overall, individual, and subgroup levels. To show the structural pattern of the network, this paper mapped the correlation network for 2020 based on UCINET6, as shown in Figure 1. The figure shows that the spatial correlation of PM_2.5_ among Chinese provinces exhibited a complex structural pattern, with multiple connections. Linked relationships connected to a particular province differed significantly in terms of quantitative characteristics, directional characteristics, etc. The structural characteristics of the spatial linkage network can be further dissected by combining elements such as policy differences, geographical location characteristics, and the economic development of the interlinked bilateral provinces.

**Figure 2:**
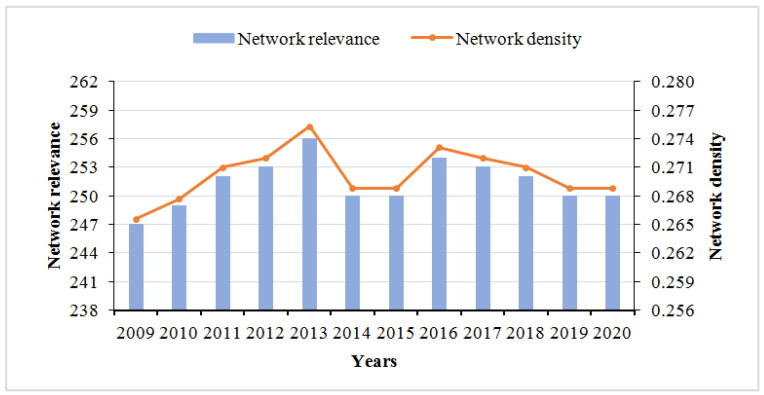
Trend of network relevance and network density from 2009 to 2020.

Figure 2 depicts the evolutionary trend of the number and density of associations in the spatial association network of PM_2.5_ in China, from 2009 to 2020. Numerically, the network association degree was relatively tight, with the maximum total number of possible relationships being 930 and the maximum actual total number of relationships being 254. In terms of fluctuating trends, the number of network relationships and the network density had obvious characteristics of fluctuating trends: they continued to rise in the 2009–2013 period but showed a significant decline in the 2013–2014 period and continued to decline after 2016. The possible reason for this is that, in 2013, China accelerated the adjustment of energy structure and implemented a new energy-saving and emission reduction mechanism that combined incentives and constraints, which significantly improved the air quality in key areas. As a result, the number of network connections and the network density decreased significantly. In 2016, China began to focus on establishing joint prevention and control mechanisms in the same regions and began strengthening PM_2.5_ monitoring and management in densely populated areas and key large provinces, thus, leading to the redundant linkages of spatially linked networks in 2016. Subsequently, there was a decreasing trend, and the closeness of PM_2.5_ among provinces also decreased with the vigorous implementation of collaborative management.

**Figure 3:**
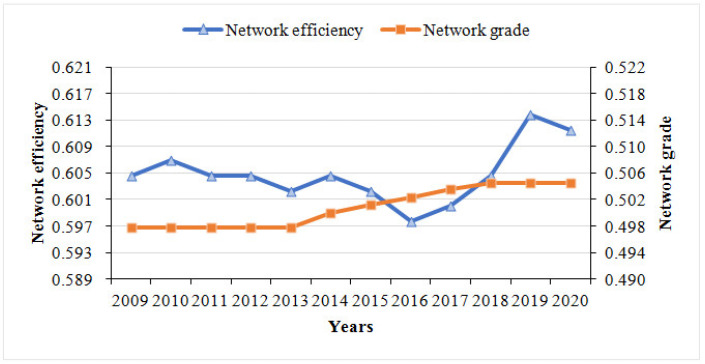
Trend of network efficiency and network rank degree from 2009 to 2020.

As presented in Figure 3, the network rank degree was generally stable and well defined. The results show that the interconnection and influence of PM_2.5_ are gradually weakening, and that the relatively clear hierarchical structure is being deconstructed. The network efficiency fluctuated between 0.5977 and 0.6138, which indicates that approximately 40% of the linkages are redundant, i.e., there are multiple superpositions of dynamic association relationships of PM_2.5_. Network efficiency showed a significant upward trend in the 2016–2020 period, indicating that network stability decreases year by year and that the network is more prone to collapse due to a break between two connections. The reason for this may lie in the supply-side reform implemented in China in late 2015, which aimed to transform the energy-driven growth mode, reduce total coal consumption, and continuously improve total factor productivity. The supply-side reform has proven to be effective, and the optimization of the energy mix can fundamentally reduce the level of haze pollution and the stability of the PM_2.5_ linkage.

### 3.2. Characteristics of the Individual Network Structure

**Figure 4:**
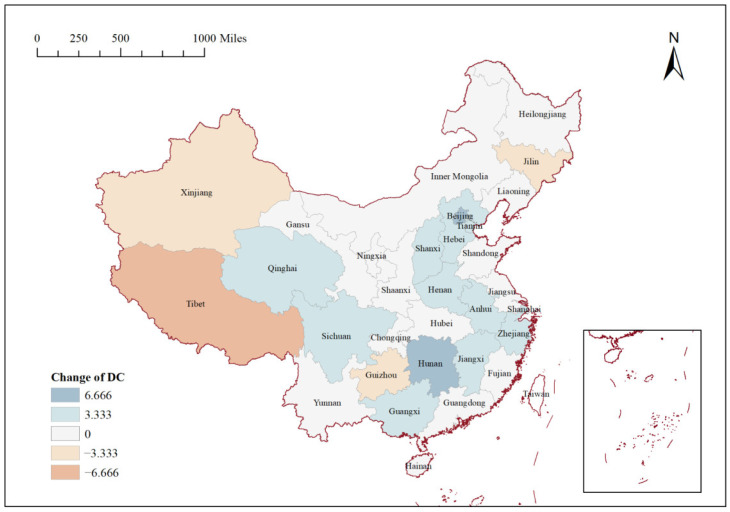
Comparison of point degree centrality by province in 2012 and 2020.

This section analyzes the individual centrality of the network by measuring DC, BC, and CC, and examines the role of each province in the network, as presented in Table 1. The comparison of the DC of each province in 2020 and 2013 was also plotted using ArcGIS software to reveal the changes in the centrality of each province in the network, due to the high concern for and control of haze pollution by the Chinese government and by society as a whole, as shown in Figure 4.

#### 3.2.1. Degree Centrality

The results in Table 1 show that the mean value of the DC of the 31 Chinese provinces in 2020 was 42.80. Thirteen provinces had a DC higher than this mean value. In descending order, they were Jiangsu, Henan, Shandong, Hubei, Guangdong, Hunan, Shaanxi, Sichuan, Chongqing, Xinjiang, Hebei, Gansu, and Tibet. These provinces had a large number of relationships with other provinces in the network. The DC of the Jiangsu, Henan, and Shandong provinces was much higher than that of the other provinces, indicating that these three provinces were at the core of the network. The Yangtze River Delta, the Beijing–Tianjin–Hebei region, and the Fenway Plain are the three key areas of concern for haze pollution management in China. Jiangsu, which is located in the Yangtze River Delta, is not only the largest province in China, in economic terms, but also the province with the most serious pollution load per unit of industry, and the highest value added of the secondary industry. In addition, the rapid economic development of Jiangsu relies on energy consumption, and the elasticity coefficient of electricity consumption in all years has been significantly higher than the national elasticity consumption coefficient of electricity. This result means that Jiangsu has higher energy dependence, energy consumption, and resource dependence on other provinces, thus, has a high DC and Ind. Shandong and Henan have the second and third largest populations in China, respectively, and their large population pressure and production and development patterns, which rely on high pollution and high emissions, exacerbate the diffusion of PM_2.5_, with obvious spatially related spillover effects. In addition, although Henan has the third largest population in China, statistics show that, as of 2019, the cumulative number of green patent applications in Henan ranked only 14th in China. Furthermore, as a latecomer, Henan has undertaken a large number of polluting enterprises that have transferred from the east in recent years, and the development of the local economy has become the primary task. In addition, the R&D investment intensity in Henan in 2019 was only 1.5%, which was far lower than the national average of 2.2%. It can be seen that the government’s support for technological innovation is insufficient. Therefore, the lack of attention to scientific and technological innovation related to air pollution is one of the reasons for its high DC.

According to the results shown in Table 1, in terms of Out, 14 provinces, such as Xinjiang, were above the mean value of 8.06. These provinces mainly showed the spatial spillover of PM_2.5_ pollution in the network. In terms of Ind, 13 provinces, including Jiangsu, were higher than the average value of 8.06, and they were mainly the recipients of the spatial spillover of PM_2.5_ pollution. For example, Zhejiang and Anhui, which are also part of the Yangtze River Delta, had a much higher Ind than Out because Zhejiang and Anhui have a “siphon effect” in the spatially linked network due to their advantages in geography, talent, and other factors. Additionally, their rapid development requires the clustering of industrial enterprises in surrounding provinces to provide resources to support them, which inevitably causes air pollution under existing conditions. Consequently, Zhejiang and Anhui play a strong role in driving PM_2.5_ pollution in other provinces. Overall, provinces that are rich in energy but relatively underdeveloped in terms of their economy and manufacturing generally corresponded to a net inflow of PM_2.5_, while provinces with a strong population and an advanced economy and industry demonstrated a net outflow of PM_2.5_.

#### 3.2.2. Betweenness Centrality

The mean BC value of the 31 provinces in 2020 was 2.047. Six provinces had a BC higher than this mean value. From high to low, they were Jiangsu, Shandong, Henan, Guangdong, Hubei, and Hunan. They had a stronger ability to control the PM_2.5_ pollution exchange among other provinces in the network. The total BC of the network was 63.446, while the total BC of the top six provinces was 47.542, representing approximately 75% of the total. This result indicates that the BC of each province showed a more obvious nonequilibrium characteristic, and the dominance and control of these six provinces over the network was higher than that of the other 25 provinces. The possible reasons for this result are as follows: First, these provinces are the main demanders and allocators of capital, talent, and resources from the surrounding areas, and their GDP and population are among the highest in China. However, they also face more serious air pollution problems. Second, the geographical locations of these provinces are either in the developed coastal regions of China, such as Jiangsu, Shandong, and Guangdong, or in the central regions, such as Henan, Hubei, and Hunan. Such locations give these provinces the geographical convenience to “communicate” with other provinces, which is conducive to better playing the role of “intermediaries”. In the process of PM_2.5_ pollution control, we should focus on “hubs” with high BC, and by controlling the PM_2.5_ emissions in these provinces, we can block the PM_2.5_ correlation between other provinces and, thus, control the PM_2.5_ spillover effect.

#### 3.2.3. Closeness Centrality

The CC measurement results presented in Table 1 show that the mean value of CC of the 31 provinces in China in 2020 was 63.99. There were 11 provinces with a CC value higher than this mean value. From high to low, they were Jiangsu, Henan, Shandong, Hubei, Guangdong, Hunan, Chongqing, Sichuan, Shaanxi, Xinjiang, and Gansu. These provinces were connected to other nodes in the network through shorter paths and could be more quickly and intrinsically connected to other provinces. The CC of Jiangsu, Shandong, and Henan was much higher than that of the other provinces. These three provinces have a higher economic level, a larger population, and a higher technological development level, and they can easily establish linkages with surrounding provinces through industrial transfer and technological spillovers, playing the role of “central actors” in the network. Other provinces are the closest to these three provinces—in other words, if these three provinces are missing, the convenience of communication among other nodes will be greatly reduced. The provinces that are less close to the center play the role of “marginal actors” in the network.

#### 3.2.4. Evolutionary Trend

This section compares the DC of each province nationwide in 2013 with that in 2020, aiming to examine the changes in the centrality and status of each province under higher policy pressure. The comparison results are shown in Figure 4. The DC of 13 provinces decreased to different degrees in 2020. Among them, the DC of Beijing and Hunan decreased by 6.666, the DC of the other 11 provinces decreased by 3.333, but the DC of Jilin, Guizhou, Tibet, and Xinjiang increased. In fact, the network correlations and BC of Beijing, Tianjin, and Hebei all showed significant decreases, in different degrees from 2016 to 2020, indicating that the spatial correlation of PM_2.5_ and its ability to act as a pollution “bridge” between the Beijing–Tianjin–Hebei region and other provinces is weakening. Although Figure 4 shows an increase in the connectivity of some remote provinces in the network, the effect achieved in China’s PM_2.5_ treatment is undeniably significant, with most of the key pollution regions—such as the Yangtze River Delta, the Beijing–Tianjin–Hebei region, the Sichuan–Chongqing region, and central China—all having declined in varying degrees in the correlation network. It is foreseeable that the trend of improving PM_2.5_ pollution in all provinces in China will continue as treatment efforts increase. Notably, however, some provinces with a higher point DC and more serious pollution had less or no decline in the correlation, which indicates that haze control still has a long way to go.

### 3.3. Spatial Clustering Analysis

This section analyzes the spatial clustering characteristics of 31 provinces in China in 2020, in the network based on the block model. Using the convergent correlations (CONCOR) iterative method in the UCINET software, the 31 provinces were divided into four blocks, with a segmentation depth of 2 and a concentration criterion of 0.2. The division results are shown in Table 2. Block I consisted of the following six provinces: Beijing, Tianjin, Hebei, Shanxi, Shandong, and Henan. There were five provinces in Block II, as follows: Jilin, Inner Mongolia, Liaoning, Ningxia, and Heilongjiang. There were nine provinces in Block III, as follows: Zhejiang, Jiangsu, Jiangxi, Anhui, Fujian, Hubei, Hunan, Guangdong, and Shanghai. Lastly, there were 11 provinces in Block IV, as follows: Hainan, Chongqing, Guangxi, Guizhou, Yunnan, Tibet, Shaanxi, Gansu, Qinghai, Sichuan, and Xinjiang.

Based on the proportion of internal linkages, external linkages, internal–external interactions, and on the actual–desired relationship, the network was divided into “net benefits”, “net spillovers”, “two-way spillovers”, and “brokers”. There were 250 correlations in the overall network, while there were 120 correlations within blocks, and 130 correlations between the blocks, which indicated that there was a more obvious spatial correlation between the blocks. Specifically, in Block I, the total number of received relationships was 90: there were 34 sent relationships and 64 received relationships outside the block. Not only did Block I have a much larger number of received relationships than the number of received relationships outside the block than the other blocks, but the actual proportion of internal relationships was also much higher than the proportion of the theoretical internal relationships. Therefore, Block I was a “net benefit” block. Block II contained six received relationships and 42 sent relationships, thus, Block II was a “net spillover” block. In Block III, the total number of received relationships was 108, that of sent relationships was 66, and the number of relationships that were internal to the block was relatively high, at 50. Despite an expected internal ratio of 30%, the block’s actual internal ratio was 76%. According to the definition in the previous section, Block III was a “two-way spillover” block. Block IV contained 46 received relationships and 108 sent relationships. 39 relationships were internal to the block, and there was a relatively high number between the block and the other blocks. Despite an expected internal ratio of 37%, the block’s actual internal ratio was 36%. According to the previous definition, Block IV was a “broker” block.

**Figure 5:**
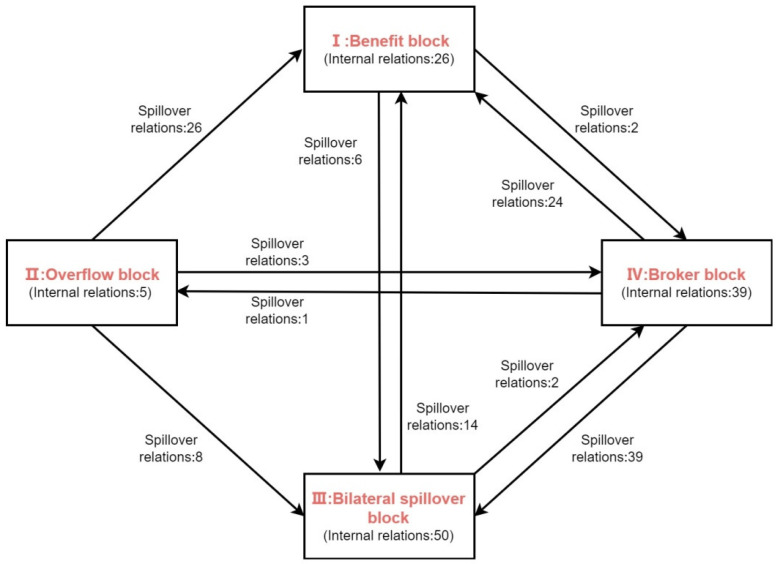
Correlation between the four blocks.

To further reveal the interrelationships of PM_2.5_ within and between blocks, the network density matrix of each block was given, as shown in Table 3. Meanwhile, based on the previous measurement, the overall network density in 2020 was 0.2688, and the network density of a block was one if it was above the overall network density, and zero if it was below the overall network density. Thus, the overall density was used as the boundary to transform the multivalue density matrix into a similarity matrix, as presented in Table 4. Figure 5 visually depicts the correlations among the four blocks, and the division of each block has obvious geographical location characteristics. The main provinces in Block I were the four provinces in the Bohai Sea region and the two central provinces adjacent to its location, which received a large number of spillover relationships from the other blocks. The geographical location of this block is “surrounded” by the other three blocks, and the unreasonable energy and industrial structures determined the tendency of PM_2.5_ to be concentrated in this block. The main provinces in Block III were the developed provinces in the coastal area and the central provinces. The provinces have a dense population and certain resource reserves, and their PM_2.5_ correlations have many relationships with the other three blocks, which are also influenced by other provinces. However, the mutual influence among the provinces within this block was more prominent. The spillover relationships of the provinces in Blocks II and IV accounted for more than 80% of the total spillover relationships, and the provinces within the block are rich in coal, oil, and natural gas. They provide many energy resources for the eastern region and are the backbone of the rapid development of the developed regions of China.

### 3.4. Factors Influencing the Network

#### 3.4.1. QAP Correlation Analysis

The QAP correlation analysis was used to test the influencing factors of the spatial association network of PM_2.5_, and the factors that passed the correlation test were considered explanatory variables in the QAP regression analysis. Based on previous studies, six indicators were selected to describe the differences between the provinces in this paper, as follows: differences in the economic development (GDP), differences in technological innovation (TI), differences in government intervention (GOV), differences in environmental regulations (ER), differences in the industrial structure (IS), and differences in car ownership (CO). The difference matrix was constructed in this way. In addition, the GD between two provinces was used to construct the geographical adjacency matrix. This paper selected 5000 as the number of random permutations. That is, the matrix rows and columns were permuted simultaneously to obtain enough correlation data, and then descriptive statistics were obtained. The magnitude of the correlation coefficient reflected the magnitude of the influence of the variable on the network structure. The maximum value and the minimum value, respectively, occurred during 5000 repeated sampling, respectively, and *p* ≥ 0 and *p* ≤ 0 were the probabilities that the correlation coefficients obtained in each iteration of the sampling process were greater than, less than, or equal to the final correlation coefficient, respectively. As shown in Table 5, the coefficients of GD, GOV, ER, IS, and CO are significant. These results indicate that these five explanatory variables were all factors affecting the spatial correlation of PM_2.5_. However, there were still strong correlations among these five explanatory variables. For example, the correlation coefficient between government intervention and car ownership was 0.292 and significant at the 1% level. If the traditional linear regression method was continued, it may have led to pseudoregression. Therefore, to analyze the influencing factors, the QAP method was used.

#### 3.4.2. QAP Regression Analysis

As presented in Table 6, the adjusted R^2^ calculated after 10,000 random permutations was 0.259, which indicated that the differences in the geographical distance, car ownership, industrial structure, environmental regulation, and government intervention explained approximately 25.9% of the variation in the network. The coefficient of GD was positive and significant at the 1%, showing that the closer the provinces were to each other, the more likely the PM_2.5_ association was. CO’s coefficient was negative and significant at the 1%, showing that the similar rate of car ownership between provinces is conducive to the formation of the network. Vehicle emissions are the raw material for PM_2.5_. Furthermore, car ownership is usually applied to measure the population and economic development level of a region, and the similar rate of civilian car ownership between regions represents similar air pollution spillovers, strengthening the network. The coefficient of IS was positive and significant at the 5%, showing that the variation in the industrial structure between regions was beneficial for the formation of the network. When industrial transfer between regions occurs, it is often accompanied by the transfer of resources and capital, thus, promoting the formation of the network. The coefficient of ER was negative and significant at the 10%, showing that the variation in environmental regulation between regions can hinder the formation of the network. The coefficient of GOV was positive and significant at the 10%, showing that the convergence of government intervention between regions was not conducive to the formation of the network. The possible reason is that, on the one hand, when there is a large difference in the intensity of government intervention between regions, PM_2.5_ pollution sources tend to shift from regions with high intervention to regions with low intervention. On the other hand, when the government intervention is stronger, the region generally has higher technology and sufficient economic support to control environmental pollution, thus, increasing the difference with the neighboring regions.

## 4. Conclusions

In this paper, we used an improved gravity model to identify the spatial association network of PM_2.5_ in 31 provinces in China, analyzed the structure of the network using SNA, and examined its influencing factors. The conclusions are as follows:

(1) The spatial association relationship of PM_2.5_ in China exhibited a more complex network structure. The network as a whole had strong stability, and there was an external spillover effect of PM_2.5_ in all provinces. In fact, with the improvement in citizens’ awareness of air pollution protection and the increase in governmental efforts, the interconnection and mutual influence of PM_2.5_ among provinces is weakening, and the relatively distinct hierarchical structure of PM_2.5_ among provinces is being deconstructed. However, it should also be realized that the network degree is more closely linked, and the current air pollution problem in China is still severe.
(2) The centrality analysis showed that the network exhibits a significant “core-edge” distribution pattern. Jiangsu, Shandong, and Henan are located at the center of the network, and these three provinces had the most connections with other provinces, and they also acted as bridges within the network. In addition, comparing the DC of each province in 2013 with that in 2020, we found that the effect of PM_2.5_ management in China was more significant, and most of the haze focus areas, such as the Yangtze River Delta, the Beijing–Tianjin–Hebei region, the Sichuan–Chongqing region, and central China, decreased to different degrees in the associated networks.
(3) A block model analysis showed that six provinces, such as Beijing, belong to the “net benefit” block, receiving a large number of spillover relationships from other blocks; five provinces, such as Jilin, belong to the “net spillover” block, sending a large number of relationships; and nine provinces belong to the “two-way spillovers” block, which plays a leading role outside the block, and the relationship within the block is also complex, which is the most difficult area for PM_2.5_ governance; and the other 11 provinces belong to the “broker” block and play a key role in the network.
(4) The results of the QAP regression analysis showed that differences in the geographical distance, car ownership, industrial structure, environmental regulation, and government intervention explained approximately 25.9% of the variation in the network among the Chinese provinces. Among them, the closer the provinces were, the more likely they were to generate the networks. Differences in government intervention and industrial structure also promotes the formation of the network, whereas differences in environmental regulation and car ownership have the opposite effect.

In view of the scientific and explanatory power of the analytical method, in future research, this model and method can also be used to study the components of PM_2.5_, such as NOx, SO_2_, CO, etc., to explore the spatial association characteristics of air pollution from a microscopic perspective. Thus, more specific and feasible suggestions could be made for pollution control.

## Figures and Tables

**Table 1:** Network centrality analysis.

Province	In-Degree	Out-Degree	Degree	Betweenness	Closeness
Beijing	11	4	36.667	0.807	57.692
Tianjin	8	3	26.667	0.23	53.571
Hebei	14	5	46.667	1.943	61.224
Shanxi	7	7	33.333	0.69	60
Inner Mongoria	0	7	23.333	0.272	56.604
Liaoning	2	8	30	0.706	58.824
Jilin	2	8	26.667	0.272	57.692
Heilongjiang	1	9	30	0.706	58.824
Shanghai	4	3	13.333	0	50.847
Jiangsu	27	6	90	17.409	90.909
Zhejiang	12	3	40	1.825	62.5
Anhui	12	7	40	0.915	62.5
Fujian	3	10	33.333	0.497	60
Jiangxi	5	9	30	0.042	58.824
Shandong	25	7	83.333	11.24	85.714
Henan	25	8	83.333	9.588	85.714
Hubei	18	8	66.667	3.077	75
Hunan	13	9	60	2.837	71.429
Guangdong	14	11	63.333	3.391	73.171
Guangxi	4	7	26.667	0.23	54.545
Hainan	0	4	13.333	0	50.847
Chongqing	9	9	50	0.847	66.667
Sichuan	9	10	50	0.707	66.667
Guizhou	6	9	33.333	0.114	58.824
Yunnan	4	10	36.667	0.225	61.224
Tibet	0	13	43.333	0.496	63.83
Shaanxi	9	10	50	1.17	66.667
Gansu	4	11	46.667	0.805	65.217
Qinghai	0	11	36.667	0.246	61.224
Ningxia	1	10	33.333	0.46	60
Xinjiang	1	14	50	1.699	66.667

**Table 2:** Analysis of spillover effects.

Block	Receive Relationship		Overflow Relationship		Expected Internal Relationship Ratio	Actual Internal Relationship Ratio
	Inside	Outside	Inside	Outside		
I	26	64	26	8	17	76
II	5	1	5	37	13	12
III	50	58	50	16	30	76
IV	39	7	39	69	37	36

**Table 3:** Subgroup density matrix.

Block	I	II	III	IV
I	0.867	0.000	0.111	0.000
II	0.867	0.250	0.178	0.055
III	0.259	0.000	0.694	0.020
IV	0.364	0.018	0.444	0.355

**Table 4:** Similarity matrix.

Block	I	II	III	IV
I	1	0	0	0
II	1	0	0	0
III	0	0	1	0
IV	1	0	1	1

**Table 5:** Results of the QAP correlation analysis.

Variable	Coefficients	Sig.	Average	Std Dev	Minimum	Maximum	*p* ≥ 0	*p* ≤ 0
GD	0.459	0.000	0.001	0.036	−0.140	0.139	0.000	1.000
GDP	0.026	0.358	0.000	0.060	−0.224	0.216	0.358	0.671
TI	−0.068	0.155	−0.001	0.063	−0.222	0.191	0.867	0.155
GOV	0.095	0.064	−0.001	0.063	−0.201	0.198	0.064	0.946
ER	−0.103	0.044	−0.001	0.057	−0.216	0.213	0.965	0.044
IS	0.168	0.002	0.000	0.059	−0.182	0.207	0.002	0.999
CO	−0.164	0.003	0.001	0.061	−0.189	0.189	0.998	0.003

**Table 6:** Results of QAP regression analysis.

Variable	Un-Standardized Coefficient	Standardized Coefficient	Significance	*p* ≥ 0	*p* ≤ 0
GD	0.555	0.443	0.000	0.000	1.000
GOV	0.075	0.080	0.062	0.062	0.938
ER	−0.069	−0.075	0.055	0.946	0.055
IS	0.075	0.082	0.040	0.040	0.960
CO	−0.165	−0.180	0.002	0.999	0.002
R^2^	0.262(0.259)				

## Data Availability

The datasets of this paper are available from the corresponding author on reasonable request.
